# Enterococci—Involvement in Pathogenesis and Therapeutic Potential in Cancer Treatment: A Mini-Review

**DOI:** 10.3390/pathogens11060687

**Published:** 2022-06-15

**Authors:** Anna Grenda, Tomasz Grenda, Piotr Domaradzki, Krzysztof Kwiatek

**Affiliations:** 1 Department of Pneumonology, Oncology and Allergology, Medical University in Lublin, Jaczewskiego 8, 20-950 Lublin, Poland; 2 Department of Hygiene of Animal Feeding Stuffs, National Veterinary Research Institute, Partyzantow 57, 24-100 Pulawy, Poland; tomasz.grenda@piwet.pulawy.pl (T.G.); kwiatekk@piwet.pulawy.pl (K.K.); 3 Department of Commodity Science and Animal Raw Materials Processing, University of Life Sciences in Lublin, Akademicka 13, 20-950 Lublin, Poland; piotr.domaradzki@up.lublin.pl

**Keywords:** *Enterococcus*, cancer, bacteriocin, bacterial metabolites

## Abstract

*Enterococcus* spp. are Gram-positive, heterogeneous lactic acid bacteria inhabiting various environments. Several species of Enterococci are considered to be able to stimulate the immune system and play an important role in intestinal homeostasis. Some Enterococci can be used as probiotics. Some strains of *E. faecium* are components of pharmaceutical products used to treat diarrhea, antibiotic-associated diarrhea, or irritable bowel syndrome (IBS). However, it has been proved that they are responsible for food contamination, and are sometimes undesirable from the point of view of food technology. Additionally, the virulence and multi-drug resistance of Enterococci potentially pose a risk of an epidemic, especially in hospital environments. Moreover, there are indications of their negative role in colon tumorigenesis; however, some nterococci are proved to support immunotherapy in cancer treatment. In general, it can be concluded that this group of microorganisms, despite its nature, has properties that can be used to support cancer treatment—both aggressive chemotherapy and cutting-edge therapy targeting immune checkpoints (IC).

## 1. Introduction

According to multiple complex human microbiome analyses, the human body is associated with approximately 5000 species of commensal microorganisms. It is estimated that they belong to approximately 2000 genera and approximately 25 phyla. This fact results in an enormous genetic diversity of approximately 316 million genes[1]. Among the mentioned and substantial number of genes, approximately nine million of them are linked with bacterial inhabitants of the human digestive tract [2]. According to the data from the Gene Catalog (IGC) of the human gut microbiome [3], nearly 40% of the mentioned genes are unknown or have functions which remain undiscovered.

*Enterococcus* spp. are Gram-positive, heterogeneous lactic acid bacteria that inhabit various environments, including the digestive tracts of humans, animals, and insects, as well as natural niches such as soil [4,5], water [6,7], sewage [8], and some kinds of plants [9,10,11]. Several species of Enterococci are considered to be able to stimulate the immune system and play an important role in intestinal homeostasis [12,13]. Some Enterococci can be used as probiotics. Some strains of *Enterococcus faecium* are components of pharmaceutical products used to treat diarrhea, antibiotic-associated diarrhea, or irritable bowel syndrome (IBS), in order to lower cholesterol levels or to improve immunity [14]. Enterococci are also applied in food technology as a starter culture triggering the fermentation process of meat and cheese, and to preserve food [15,16,17]. Enterococci can also be recognized as pathogens [18]. Especially in nosocomial infections, enterococci (particularly *Enterococcus faecalis* and *Enterococcus faecium*) play a significant role. They can cause intra-abdominal and urinary tract infections, bacteremia, and endocarditis [19]. They are responsible for food contamination, and are sometimes undesirable from the point of view of food technology [16]. Additionally, the virulence and multi-drug resistance of Enterococci potentially pose a risk of an epidemic, especially in hospital environments [20]. Recently conducted research indicated their negative role in colon tumorigenesis [21]. 

Moreover, *E. faecalis* has been described as a bacterium involved in colorectal cancer due to the fact that its metabolic products can damage the DNA of intestinal epithelial cells, which may contribute to the formation of neoplasia, and may even disrupt the expression of genes responsible for repairing DNA damage, e.g., PMS1 (PMS1 Homolog 1, Mismatch Repair System Component), MSH2 (MutS Homolog 2), MSH3 (MutS Homolog 3), MSH6 (MutS Homolog 6), and controlling the cell cycle [22]. Wang and Huycke indicate that *E. faecalis* produces superoxide, which can lead to chromosomal instability in eukaryotic cells [23]. Huycke et al. demonstrated that chromosomal instability in colon cancer cells is promoted by reactive oxygen species secreted extracellularly by *E. faecalis* [24].

On the other hand, enterococci are proved to support immunotherapy in cancer treatment [25]. There are now a growing number of reports on the role of Enterococci in cancer treatment. These bacteria produce a whole range of substances that have antimicrobial and/or anticancer properties. More and more research is emerging on this synergism of action (bactericidal and tumoricidal in some cases), which can be exploited in the treatment of immunocompromised cancer patients exposed to frequent life-threatening bacterial infections, first as substances to control the infection, but at the same time as an adjuvant in basic oncological treatment. In addition, certain bacterial strains of *Enterococcus* spp. can induce or promote the action of the lymphocytes responsible for recognizing and destroying cancer cells (Table 1).

The diversity and activity of Enterococci in different medical conditions are not fully elucidated. Some reports disregard enterococci regardless of their positive features; however, an increasing number of studies are appearing regarding their therapeutic potential and elucidating their hazardous activity in certain situations. The regular development of new metagenomic tools is changing our understanding of the human microbiome and its diversity. Metagenomic analyses provide us with the necessary data to understand how the gut microbiota influences our health [26]. The aim of this study is to review the latest reports on the therapeutic and pathogenic potential of Enterococci in oncology.

## 2. *Enterococcus faecalis* and Colorectal Cancer

Colorectal cancer (CRC) is one of the most frequently diagnosed cancers in the world [27]. One of the potential reasons for the increasing number of CRC cases, especially in developing countries, is associated with changes in lifestyle [28,29]. This association is supported by the knowledge that only 15% of CRC cases are due to heritable genetic mutation. Approximately 85% of cases possess a sporadic character [27]. Additionally, environmental factors are suspected to be involved in CRC pathogenesis, such as: obesity, alcoholism, smoking, diabetes mellitus, sedentary lifestyle, excessive red meat consumption, inadequate intake of fiber, and a high-fat diet [30]. The gut microbial composition has also been reported as a very important condition linked with the progress of CRC [31].

The development of metagenomic tools is enhancing the research showing modulation of the mucosal immune system and changes to the expression of some host genes associated with important functions ensured by gut microorganisms. These functions should be included: nutrient uptake, metabolism, angiogenesis, and mucosal barrier [32]. The imbalance of the intestinal microbiota could break the integrity of the intestinal epithelial, causing serious repercussions such as inflammation, oncogenesis, and the progression of primary tumors into metastasis [14].

As a result of damage to the intestinal wall, *E. faecalis*, which is part of the normal intestinal flora, can escape through the broken wall and cause systemic infections such as infective endocarditis [33]. In many patients with endocarditis caused by *E. faecalis*, in identified origin cases, the source is genitourinary [33,34]. However, in most cases, the source of *E. faecalis* is unidentified [34]. Khan et al. indicated that in patients with an unidentified source, colonoscopy may reveal hidden early-stage CRC or adenoma [33]. Khan et al. [33] suggested that in cases of bacteremia caused by *E. faecalis* and endocarditis of an undetermined source, colonoscopy should be considered, if possible, to rule out a diagnosis of colorectal cancer. Pericàs et al. [34] conducted a study among 154 patients with definite *E. faecalis* infective endocarditis (EFIE). In total, forty-five had a confirmed source of infection (genitourinary tract, catheter, surgical wound, biliary tract origins), and 109 had an unclear source of infection to check the prevalence of colorectal neoplasms (CRN) and colorectal diseases. In the group with an unknown source of infection, 56% of patients underwent colonoscopy and half of them were found to have a colorectal neoplasm. Researchers such as Khan et al. indicate that colonoscopy should be performed in patients with EFIE and an unclear source of infection [34]. Interesting observations were made on the role of *E. faecalis* in CRC formation and its progression by Williamson et al. [35]. They found that the presence of collagenolytic bacteria, such as *E. faecalis* in the colon at the site of tumor removal, especially at the site of anastomosis, was associated with tumor progression. Studies on mouse models confirmed that the presence of this bacterium was associated with the induction of invasion and migration of neoplastic cells, and the collagenolytic abilities of *E. faecalis* played a significant role in the invasion process. They also observed that g*elE*, a gene required for collagenase production, is expressed in *E. faecalis* in response to contact with murine colorectal carcinoma cell lines [35]. This result suggests that GelE produced by *E. faecalis* may assist in the release of cancer cells from intracellular matrix after surgery and thus make it easier for them to exit and translocate to distal sites. The researchers also postulate that *E. faecalis* promotes the migration of cancer cells through its ability to activate the pro-uPA component of the urokinase–plasminogen system, a pathway that is well-known to be important in the invasion and migration of cancer cells [35]. The study suggests that the clinicians should consider Enterococcus bacteremia as a potential manifestation of colon cancer, especially in patients without a clear source of bacteremia [36]. *E. faecalis* is the best-described Enterococcus contributing to colorectal cancer formation and progression; however, Zamora and colleagues also point to *E. faecium* as a potential factor in colorectal cancer formation [36].

## 3. Therapeutic Potential of Enterococci and Its Metabolites

*E. faecalis* migration and infection have been implicated in the pathogenesis of cancer [35]. However, it begs the question of what comes first—the tumor in the intestine or the inflammation and microdamage to the intestinal wall by the bacteria that contributes to tumor formation. On one hand, *E. faecalis* produces reactive oxygen species that can cause neoplasia by damaging genomic and mitochondrial DNA; on the other hand, *Enteroccocus* spp. produce a range of substances that enable them to live in the gut and compete with other microorganisms [24]. Perhaps maintaining the homeostasis of the gut, and thus the production of microbial substances, helps to prevent cancer. It has been shown that the formation of cancer is associated with a disruption in the composition and quantity of microorganisms in the gut (alpha and beta diversity), thus changing the composition of the bacteriocinome, which includes a number of peptides secreted by almost all groups of bacteria that have antimicrobial properties as well as the potential to eliminate cancer cells [24,37,38,39,40]. 

Bhalla et al. indicated that dysbiosis contributes to the development of colon cancer, and that an effective treatment for this disease can be found in microorganisms [41]. They focused on *Enterococcus* spp. bacteria, which exhibit probiotic and antineoplastic properties [41]. The authors postulate that treatment based on induced reactive oxygen species by nanoparticles may be effective in CRC, by modulating the secretion of anticancer substances, and the anticancer potential of *Enterococcus durans* was tested. They treated a culture of this bacteria with silver nanoparticles (AgNPs), which led to an increase in the extracellular folate concentration. Based on a computational study, the researchers indicate certain metabolic pathways involving amino acids, energy metabolites, nucleotides, and short-chain fatty acids as the key players in elevating folate levels on ROS exposure [41]. Moreover, the researchers decided to test the anti-neoplastic properties of *E. durans* on 116 HCT cell lines supplemented with culture supernatants of this bacterium treated with AgNPs. The results show that the nanoparticles most likely stimulate the production of metabolites, leading to cancer cell death, and the viability of the cancer cells treated with the supernatant from the nanoparticle-treated cultures was reduced by 19% as compared to a control [41]. Bacteria and their metabolites are increasingly being studied for their importance in oncology. Examples of bacteria that may be important in anticancer therapies are shown in Table 1.

**Table 1:** Potential of enterococci as anticancer therapies.

Enterococcus Strain	Characteristics	Literature
*Enterococcus durans*	AgNP-treated bacterial culture supernatant with cytotoxic effect against CRC cells, associated with folic acid metabolism.	[41]
*Enterococcus faecium 12a*	Metabolites with properties that inhibit multidrug-resistant strains of pathogens, such as *Salmonella enterica*, *Shigella flexneri*, *Vibrio cholerae*, *Escherichia coli*, and *Listeria monocytogenes* by producing enterocin 12a. - Selectively inhibits the proliferation of cervical cancer, CRC, and lung cancer cells.	[42]
*Enterococcus thailandicus*	Inhibition of hepatocellular carcinoma cell growth by enterocin LNS18.	[43]
*Enterococcus mundtii C4L10*	Apoptogenic, antimicrobial, and antiproliferative properties of bacteriocin 10 kDa in size, in breast cancer, lung cancer, and colon cancer cells.	[44]
*Enterococcus. faecalis 16H*	Metabolites exhibiting apoptotic effects against gastric cancer, cervical cancer, breast cancer, and colon adenocarcinoma cells.	[45]
*Enterococcus faecalis LD33 (aerobically cultured)*	Bacteria and supernatant: - Inhibitory effect on the growth of colon adenocarcinoma cells. - Antimicrobial properties against *Listeria monocytogenes* and *Staphylococcus aureus*.	[46]
*Enterococcus hirae* *Enterococcus faecium*	Metabolites with: - Antimicrobial activity against *Mycobacterium smegmatis*. - Inhibition of tumor cell proliferation of cervical cancer, CRC, and lung cancer cells. Ability to remodel peptidoglycan and support immunotherapy using immune checkpoint inhibitors.	[25,42,47,48,49,50]
*Enterococcus faecium M-74*	Prevent the occurrence of neutropenic fever in patients with solid and hematologic malignancies.	[51]
*Enterococcus casseliflavus*	Potential biomarkers for chemotherapy responses in lung cancer patients.	[52]

Bacterial metabolites of enterococcus origin in anticancer treatment are indicated by Sharma et al. [42]. As there are an increasing number of Gram-negative bacterial infections in neutropenic patients, dual-action anticancer and antimicrobial activity therapies are being sought. The researchers decided to test bacteriocins from the supernatant of *E. faecium* 12a cell culture. It was found that Enterocin 12a inhibited multidrug-resistant strains of various Gram-negative pathogens, such as *Salmonella enterica*, *Shigella flexneri*, *Vibrio cholerae* and *Escherichia coli*, and Gram-positive *Listeria monocytogenes,* but had no activity against different strains of gut lactobacilli and simultaneously selectively inhibited the proliferation of various human cancer cell lines in a dose-dependent manner but not that of normal human peripheral blood mononuclear cells [42]. Tumor cells treated with enterocin-12a showed morphological changes similar to those that occur during apoptosis [42]. Al-Madboly et al. conducted a study looking for bacteriocins with anticarcinogenic properties in fermented dairy products. They isolated thirty *Enterococcus* spp. strains from these products, and found that ten *E. faecalis*, five *E. faecium*, eight *E. durans*, two *Enterococcus thailandicus*, and four *E. avium* had antibacterial properties. After the analyses, they chose *E. thailandicus* for further study [43]. They studied the bacteriocins that these bacteria can secrete against the HepG2 cell line. They observed that enterocin LNS18 showed the highest anticancer effects against HepG2 cells with 75.24% cellular inhibition, and no cytotoxic effects on normal fibroblast cells [43]. The identified enterocin shared around 95% identity with enterocin NKR-5-3B from *E. faecium*. The researchers indicate that this enterocin is a potential liver-cancer-cell-destroying agent and shows no toxicity to normal cell lines [43]. The *Enterococcus mundtii* C4L10 strain has apoptogenic, antimicrobial, and antiproliferative properties. The investigation led by Moshood et al. indicates that a bacteriocin of the approximate size of 10 kDa produced by *Enterococcus mundtii* C4L10 can be used as a potential antimicrobial agent and as an anticancer agent because it exhibits antiproliferative properties against breast cancer, lung cancer, and colon cancer cell lines [44].

The anticancer potential of *E. faecalis* 16H is indicated by Nami et al. [45]. The authors point out acceptable apoptotic effects on the AGS, HeLa, MCF-7, and HT-29 human cancer cell lines with negligible side effects on the assayed non-cancerous (HUVEC) cell line. They suggest that metabolites of the newly isolated *E. faecalis* (confirmed by NGS—next-generation sequencing) have pro-apoptotic properties. However, they do not indicate what kind of metabolites could have caused the apoptosis [45]. This study is interesting, but requires further research in distinguishing the metabolites and their potential anticancer properties singly or in synergistic action. Jiao and colleagues investigated the overall properties of the facultative anaerobes *E. faecalis* LD33 cultured under conditions focused on aerobic metabolism in an anti-bactericidal and anticancer context [46]. The bacteria cultured under aerobic conditions showed antimicrobial properties against *Listeria monocytogenes* ATCC19111 and *Staphylococcus aureus* ATCC6538P. The properties of the supernatant showed a stronger inhibitory effect on the growth of tumor cell line HT-29 cells than the live bacteria and was comparable to the effect of 5-Fluorouracil (7 μg/mL) [46]. In general, aerobically cultured *E. faecalis* LD33 produced more substances, such as acetic acid, acetoin, and diacetyl, and less lactic acid [46]. The metabolites of three enterococcus strains, *Enterococcus hirae* 20c and *Enterococcus faecium* 12a and L12b, inhibited the *in vitro* proliferation of various human cancer cell lines in a dose-dependent manner, but had no activity against normal human peripheral blood monocytes [47]. Furthermore, the studied strains had antimicrobial activity against various Gram-positive and Gram-negative pathogens, including *Mycobacterium smegmatis* [47]. All strains tested had probiotic characteristics,—showing strong tolerance to gastric juice and bile, and had the ability to adhere strongly to colonic epithelial cells (HCT-15) [47]. Sharma et al. indicate the need to conduct *in vitro* tests of *E. hirae* and *E. faecium* as probiotic bacteria with anticancer properties [47]. This type of research was carried out by Griffin et al. in a mouse cancer model [25]. It has been suggested that bacteria with the ability to remodel peptidoglycan secrete substances that have this anticancer property, and ultimately muropeptide-based (SagA-engineered probiotics or synthetic muropeptides) drugs, could be used as adjuvant therapy to support immunotherapy [25]. The effectiveness of cancer treatment with the use of therapy aimed at immunological checkpoints (anti-Cytotoxic T-Lymphocyte Associated Protein 4, anti-PD-1, and anti-Programmed Cell Death Ligand 1) may depend on the composition of the microbiome [25]. Nevertheless, the molecular mechanisms by which certain bacterial strains can support immunotherapy remain not entirely clear. It has been found that Enterococcus can support immunotherapy by secreting orthologs of NlpC/p60 peptidoglycan hydrolase SagA that generate immune-active muropeptides [25]. The researchers also indicate that *E. faecalis* is a promoter of response to immunotherapy, and its activity is strongly associated with the sensor peptidoglycan NOD2 (nucleotide-binding oligomerization domain containing 2) involved in the immune response to bacterial lipopolysaccharides (LPS) by recognizing the muramyl dipeptide (MDP) derived from them and activating the NFKB protein (Nuclear Factor Kappa B) [25].

## 4. Allies in Cancer Therapy

Enterococci can support treatment with therapeutics currently used in everyday clinical practice, both in systemic treatment and in the most modern immunotherapy.

Daillère et al. indicate that IEC NOD2 receptors can serve as ‘gut immune checkpoints’ limiting the immunogenicity of bacteria [48]. NOD2 reduces cyclophosphamide-induced tumor immune surveillance and *E. hirae* activity [48]. The researchers discuss the loss-of-function mutations of the *NOD2* gene, and found that impaired expression of NOD2 under pathological conditions facilitates the induction of an immune response against these bacteria [48]. Moreover, the researchers demonstrated that this property of *E. hirae* increases the efficacy of cyclophosphamide in dysbiosis caused by broad-spectrum antibiotics [48]. In addition, they indicated that *E. hirae* translocate from the small intestine to secondary lymphoid organs, and the increased intracellular CD8/Treg and Th1 responding to *E. hirae* were significantly associated with prolongation of PFS (progression free survival) [48]. 

After analyzing the microbiome composition of patients with metastatic skin cancer treated with anti-PD-1 immunotherapy, it was found that in patients in whom the therapy was effective, more abundant *Enterococcus faecium* was observed. The material for the research was collected before the implementation of the treatment, so it indicates that the abundance of this bacteria at baseline for treatment has an impact on the effectiveness of therapy with immune checkpoint inhibitors [49]. Furthermore, in vivo studies of germ-free mice have shown that fecal microbiome transplantation from responding patients provides improved tumor control, enhanced T-cell responses, and better efficacy of anti-PD-L1 therapy [49]. 

Cyclophosphamide has the ability to destroy immunosuppressive T cells and stimulate antitumor immune responses. Viaud et al. demonstrated in mouse models that this drug alters the composition of the microflora in the small intestine and induces the translocation of selected species of Gram-positive bacteria including *E. hirae* to secondary lymphoid organs, thus enhancing the immunomodulatory effect of cyclophosphamide due to the accumulation of the T17 lymphocytes and T1 memory cells necessary for the CTX (chemotherapy combination with cyclophosphamide)-induced antitumor immune response [50].

Mego et al. studied the effect of an *Enterococcus faecium* M-74 strain enriched with organic selenium in patients with solid and hematologic malignancies on preventing the occurrence of neutropenic fever, which remains a life-threatening complication of chemotherapy [51]. The researchers based their study on the premise that colonization of the intestine by pathogenic bacteria and breaking down the intestinal barrier is an important aspect of the development of neutropenic fever. The authors suggest that to avoid complications associated with fever, probiotics could be administered to compete with the pathogens that cause neutropenic fever. Probiotic bacteria can colonize the intestinal wall, thereby limiting the growth and migration of pathogenic bacteria. In their study, such a probiotic bacterium was *E. faecium* M-74. Deep molecular study, including molecular fingerprinting techniques, phenotypic studies, and whole-genome analysis using next-generation sequencing, indicate that strains causing nosocomial infections are genetically and phenotypically distinct. Therefore, strains with probiotic potential such as *E. faecium* M74 are being selected for testing in such a way that they are safe to use and do not cause infection [52]. An 18 × 10^9^ CFU/mL dose strain of *E. faecium* M-74 was administered to patients with germ cell tumors and relapsed acute leukemia, and no febrile episode was noted in patients with germ cell carcinoma. Tolerance of therapy overall was excellent, with no significant side effects [52]. A microbiome study of 64 intestinal stool samples at baseline in patients with locally advanced and advanced lung cancer prior to chemotherapy treatment showed that *Enterococcus casseliflavus* is more abundant in responding patients compared to non-responding patients [53]. The researchers indicate that *E. casseliflavus* can be used as a biological biomarker for chemotherapy response [53].

## 5. Conclusions

The nature of Enterococci remains problematic and not fully elucidated. Ferchichi and colleagues point out a number of difficulties and challenges in implementing enterococci as candidates for useful and beneficial applications in the food industry, food biotechnology, or medicine [54]. Establishing the harmlessness of the strain, implementing appropriate risk, and benefit analysis of Enterococci as allies are mentioned [54]. This seems to be particularly important in the aspect of oncological treatment, where the use of selected Enterococci to support oncological therapies could be effective. However, taking their nature into account, they should be used with caution, especially in immunodeficient patients.

Research is needed on the therapeutic potential of enterococci, more than likely in different ethnic groups that have different microbiome composition, but also different mutational profiles of tumors. In vitro studies could also include 3D tissue models, where the analysis of host–microbe interactions in both pathogenic and therapeutic aspects would be deepened. An important aspect will be meta-analyses, which will examine, e.g., the relationship between the pathogenic/therapeutic effects of enterococci in relation to the mutational, transcriptomic, or proteomic profile. The current techniques of high-throughput studies, such as next-generation sequencing, allow such analyses. However, they require the expertise of highly specialized molecular biologists and bioinformaticians who are able to analyze very large numbers of data obtained in such studies. Assessing the risks associated with using potential pathogens such as *E. faecalis* and *E. faecium* as therapeutic agents is critical. An in-depth understanding of the genetic diversity of individual enterococci strains will allow identification of those that are primarily safe, with potential probiotic characteristics that may benefit cancer patients.

## Data Availability

No data were created or analyzed in this study. Data sharing is not applicable to this article.
